# Body Composition Changes After Bariatric Surgery or Treatment With GLP-1 Receptor Agonists

**DOI:** 10.1001/jamanetworkopen.2025.53323

**Published:** 2026-01-09

**Authors:** Zicheng Wang, Lei Wang, Xinmeng Zhang, Brandon D. Lowery, Lauren Lee Shaffer, You Chen, Quinn S. Wells, Charles R. Flynn, Brandon Williams, Matthew Spann, Gitanjali Srivastava, Jason M. Samuels, Danxia Yu

**Affiliations:** 1 Division of Epidemiology, Department of Medicine, Vanderbilt University Medical Center, Nashville, Tennessee; 2 Department of Computer Science, Vanderbilt University, Nashville, Tennessee; 3 Vanderbilt Institute for Clinical and Translational Research, Vanderbilt University Medical Center, Nashville, Tennessee; 4 Division of Cardiovascular Medicine, Department of Medicine, Vanderbilt University Medical Center, Nashville, Tennessee; 5 Department of Biomedical Informatics, Vanderbilt University Medical Center, Nashville, Tennessee; 6 Department of Surgery, Vanderbilt University Medical Center, Nashville, Tennessee; 7 Division of Diabetes, Endocrinology and Metabolism, Department of Medicine, Vanderbilt University School of Medicine, Nashville, Tennessee; 8 Department of Pediatrics, Vanderbilt University School of Medicine, Nashville, Tennessee

## Abstract

**Importance:**

The association of bariatric surgery and newer glucagon-like peptide-1 receptor agonists (GLP-1RAs; semaglutide and tirzepatide) with body composition still lack evidence from clinical settings.

**Objective:**

To examine temporal changes in fat-free mass (FFM), fat mass (FM), and FFM to FM ratio after bariatric surgery or GLP-1RA treatment over 24 months.

**Design, Setting, and Participants:**

This retrospective cohort study used electronic health records from Vanderbilt University Medical Center of 1257 patients aged 18 to 65 years who underwent first-time bariatric surgery from November 21, 2017, to July 21, 2022, without GLP-1RA treatment from 1 year before to 2 years after surgery, and 1809 nonsurgical patients who started semaglutide or tirzepatide between November 12, 2018, and December 6, 2023, with 2 or more prescriptions or 5% or more weight loss. All patients had 2 or more bioelectrical impedance analysis measures and no history of end-stage kidney disease or congestive heart failure.

**Exposures:**

Bariatric surgery or GLP-1RA (semaglutide or tirzepatide) treatment.

**Main Outcomes and Measures:**

Relative changes in FFM, FM, and FFM to FM ratio over 24 months, controlling for age, sex, race, baseline body mass index (BMI), diabetes history, treatment year, time (restricted cubic splines), and time spline-by-treatment interaction.

**Results:**

The study comprised 3066 patients: 1257 in the surgery group (mean [SD] age, 43.4 [10.3] years; mean [SD] baseline BMI, 46.8 [7.1]; 1033 women [82.2%]) and 1809 in the GLP-1RA group (mean [SD] age, 45.4 [11.3] years; mean [SD] baseline BMI, 41.0 [7.9]; 1457 women [80.5%]). Adjusted mean relative FM reductions in the surgery group were 42.4% (95% CI, 41.5%-43.2%) at 6 months, 49.7% (95% CI, 48.8%-50.6%) at 12 months, and 49.7% (95% CI, 47.8%-51.5%) at 24 months; reductions in the GLP-1RA group were 10.3% (95% CI, 9.5%-11.0%) at 6 months, 17.3% (95% CI, 16.5%-18.1%) at 12 months, and 18.0% (95% CI, 16.4%-19.7%) at 12 months. Adjusted mean relative FFM reductions in the surgery group were 7.8% (95% CI, 7.2%-8.4%) at 6 months, 10.6% (95% CI, 10.0%-11.2%) at 12 months, and 11.7% (95% CI, 10.4%-12.9%) at 24 months; reductions in the GLP-1RA group were 1.8% (95% CI, 1.3%-2.4%) at 6 months, 3.0% (95% CI, 2.4%-3.5%) at 12 months, and 3.3% (95% CI, 2.1%-4.4%) at 24 months. FFM to FM ratios increased significantly in both groups, with surgical patients maintaining a higher ratio throughout: the FFM to FM ratios in the surgery group were 1.8 (95% CI, 1.8-1.8) at 6 months, 2.1 (95% CI, 2.1-2.1) at 12 months, and 2.0 (95% CI, 2.0-2.1) at 24 months; the FFM to FM ratios in the GLP-1RA group were 1.4 (95% CI, 1.4-1.4) at 6 months, 1.5 (95% CI, 1.4-1.5) at 12 months, and 1.5 (95% CI, 1.5-1.6) at 24 months. Similar trends were observed in stratified analyses by sex, race, baseline BMI, baseline diabetes status, and GLP-1RA treatment duration, although men showed better FFM preservation than women, especially after GLP-1RA treatment.

**Conclusions and Relevance:**

In this single-center cohort study, both bariatric surgery and semaglutide or tirzepatide treatment were associated with substantial FM loss, moderate FFM loss, and improved FFM to FM ratio. These findings provide evidence to guide interventions aimed at preserving FFM while promoting fat loss.

## Key Points

**Question:**

How are bariatric surgery and treatment with newer glucagon-like peptide-1 receptor agonists (GLP1-RAs; semaglutide and tirzepatide) associated with body composition in a clinical setting?

**Findings:**

In this single-center cohort study of 3066 patients, both bariatric surgery and GLP-1RA treatment were associated with substantial fat mass loss, modest fat-free mass loss, and increased fat-free mass to fat mass ratio over 24 months. All these changes were more evident among patients who underwent bariatric surgery.

**Meaning:**

This study suggests that both bariatric surgery and GLP-1RAs are associated with effective reduction of fat mass over 2 years, with bariatric surgery associated with a more favorable fat-free mass to fat mass ratio than GLP-1RAs due to greater fat mass loss.

## Teaser

This cohort study examines the associations of bariatric surgery or glucagon-like peptide-1 receptor agonist (GLP-1RA) treatment with body composition over 24 months.

## Introduction

Obesity is a significant public health crisis worldwide and in the US. Based on reports from 2023, 40.3% of US adults had obesity (defined as body mass index [BMI] ≥30 [calculated as weight in kilograms divided by height in meters squared]), and 9.4% had severe obesity (BMI ≥40); severe obesity had doubled since 2000.^1,2^ Projections showed that by 2030, almost half (48.9%) of US adults will have obesity and approximately 1 in 4 adults will have severe obesity.^3^ Obesity is a major risk factor for health conditions, including type 2 diabetes,^4^ hypertension,^5^ various cardiovascular diseases (CVDs),^6^ asthma,^7^ and at least 13 types of cancer.^8^ Treating obesity will help alleviate disease burden and improve quality of life.

Current obesity treatments include lifestyle, pharmacologic, and surgical interventions.^9^ Among these, bariatric surgery and the new generation of glucagon-like peptide-1 receptor agonists (GLP-1RAs) have demonstrated the most substantial effects on weight loss. Bariatric surgery, including sleeve gastrectomy and Roux-en-Y gastric bypass, could maintain 25.5% and 27.7% weight loss, respectively, over 10 years.^10^ The STEP (Semaglutide Treatment Effect in People With Obesity) 5 trial reported that semaglutide, 2.4 mg, resulted in a mean 15.2% reduction in body weight over 2 years,^11^ whereas the SURMOUNT trials showed that tirzepatide, 10 mg, led to a 12.8% to 19.5% reduction in body weight and tirzepatide, 15 mg, led to a 14.7% to 20.9% reduction in body weight at 72 weeks.^12,13^ However, the measurement of weight or BMI overlooks body composition.^14^ Results from a meta-analysis of 7 prospective cohort studies encompassing 16 155 participants showed that, while higher percentages of fat mass (FM) were associated with elevated mortality risk, having higher percentages of fat-free mass (FFM) was a protective factor against mortality.^15^ Studies also showed that among patients with coronary artery disease, a higher body fat percentage was associated with a higher risk of major adverse cardiovascular events, whereas a higher FFM was associated with a better prognosis.^16^ Other studies found that a higher FFM to FM ratio was associated with a worse prognosis of asthma and a higher risk of fibrosis.^17,18^ Therefore, it is important to monitor changes in FM and FFM during obesity treatment, aiming to reduce FM while preserving FFM.

Although evidence on changes in body weight or BMI after bariatric surgery or GLP-1RA treatment has been abundant,^19,20,21,22,23,24^ longitudinal data on body composition changes remain limited. Previous research was constrained by small sample sizes (usually <100), short follow-up times (usually <27 weeks),^25^ or limited generalizability.^26^ In addition, studies on newer GLP-1RAs and body composition changes in clinical settings, as well as comparisons with bariatric patients from the same hospital, are currently lacking. Herein, we used electronic health record (EHR) data from Vanderbilt University Medical Center (VUMC) to examine longitudinal changes in FM, FFM, and FFM to FM ratio over 24 months after bariatric surgery or treatment with newer GLP-1RAs (semaglutide or tirzepatide). Our study was not designed to directly compare 2 treatments but rather to reveal changes in body composition over time after either surgical or medical weight loss using clinical data. The findings may help guide clinical obesity care and inform strategies to improve body composition along with weight loss.

## Methods

### Study Population

This cohort study included patients who underwent first-time bariatric surgery between November 21, 2017, and July 21, 2022, and those treated with semaglutide or tirzepatide from November 12, 2018, to December 6, 2023, at VUMC. Included patients had body composition measures at baseline and at least 1 measure within 24 months after treatment initiation. Patients were excluded if they were younger than 18 years or older than 65 years or had a history of end-stage kidney disease (*International Classification of Diseases, Ninth Revision* [*ICD-9*] code 585.6 and *International Statistical Classification of Diseases and Related Health Problems, Tenth Revision* [*ICD-10*] code N18.6) or congestive heart failure (*ICD-9* code 428.0 and *ICD-10* code I50.22). For the surgery group, we further excluded those who used semaglutide or tirzepatide from 1 year before to 2 years after surgery, resulting in 1257 participants. The GLP-1RA group included 1809 patients who had at least 2 prescriptions or achieved 5% or more weight loss to identify those likely with continued medication use and good adherence (study flowchart in eFigure 1 in Supplement 1). Information on demographic characteristics (age, sex, and race), surgery (date and type of procedure), prescription (date and type of GLP-1RA), disease history (diabetes, hypertension, dyslipidemia, and medication use), and baseline BMI were obtained from the EHR. Self-reported race was grouped into Black or African American, White, and other (including American Indian or Alaska Native, Asian, Middle Eastern or North African, Native Hawaiian or Other Pacific Islander, multiracial, and unknown) due to small sample sizes. Data on race were collected in the EHR as part of routine clinical practice. Diagnosis criteria for comorbidities can be found in a previous publication.^27^ The study was approved by the VUMC institutional review board, which waived the requirement for written informed consent due to the retrospective design and minimal risk to participants. The Strengthening the Reporting of Observational Studies in Epidemiology (STROBE) reporting guideline reporting guideline was followed.^28^

### Measurement of Body Composition

Body composition was quantified by bioelectrical impedance analysis (BIA), which is a widely used, noninvasive technique for estimating FM and FFM. BIA works by transmitting a low-level electrical current through the body and measuring the impedance or resistance encountered. Based on these impedance measurements, along with individual characteristics such as height, weight, age, and sex, BIA is able to estimate FM and FFM.^29^ Although BIA is less accurate than criterion standard methods such as dual-energy x-ray absorptiometry (DXA), it provides a practical, accessible, and cost-effective tool for monitoring longitudinal changes in body composition, especially in large-scale clinical settings.^30^ For surgical patients, the baseline BIA measurement was defined as the median value during the 6 months before surgery. For patients treated with GLP-1RA, the baseline BIA measurement was defined as the assessment performed from 3 months before to 1 month after the first prescription. All patients subsequently underwent follow-up BIA measurements at multiple, irregular time points within 24 months after treatment initiation.

### Statistical Analysis

Baseline characteristics were summarized, presenting mean (SD) values for continuous variables and counts with percentages for categorical variables. Relative reductions in FM and FFM were calculated as the percentage change from each individual’s baseline value: relative reduction (%) = [(baseline value − follow-up value)/baseline value] × 100%. The FFM to FM ratio and the FFM loss percentage of total weight loss (FFML%TWL) were also calculated at each time point. Biologically implausible extreme values (eg, FFM or FM >226.8 kg [500 lb] or relative change >100%) that were likely erroneous were excluded from all analyses.

To visualize temporal changes in body composition, we presented observed mean values of body composition measures within each month. To display smoothed trends, we overlaid centered 6-month moving mean values and used a pooled-variance standard error to construct the t-based 95% CIs for the smoothed curve. Month 0 was anchored to its unsmoothed bin estimate to avoid edge distortion. For inference, adjusted generalized linear mixed models with random intercepts were used to estimate the relative changes of FFM, FM, and FFM to FM ratio at 6, 12, and 24 months after treatment. Fixed effects included treatment group, time (modeled as a restricted cubic spline with 3 or 5 knots), the interaction between treatment group and time spline, and covariates, including age, sex, race, baseline BMI, history of diabetes, and treatment year. The optimal number of knots for the time spline when modeling each outcome was determined based on smaller Akaike information criterion values. Specifically, 3 knots were placed at the 10th, 50th, and 90th percentiles for FFM to FM ratio, and 5 knots were placed at the 5th, 27.5th, 50th, 72.5th, and 95th percentiles for FM and FFM losses. Group differences at 6, 12, and 24 months were evaluated using least-squares mean value contrasts with Wald-type *t* tests. To account for multiple testing across the 3 time points, a Bonferroni correction was applied, setting the significance threshold at *P* < .02 (.05/3). Stratified analyses were conducted by sex, race, baseline BMI (≤40 or >40), diabetes status, and GLP-1RA treatment duration (<12 months or ≥12 months). Analyses were performed in R, version 4.3.3 (R Project for Statistical Computing) and SAS, version 9.4 (SAS Institute Inc).

## Results

Table 1 presents the baseline characteristics of 1257 surgical patients (mean [SD] age, 43.4 [10.3] years; mean [SD] baseline BMI, 46.8 [7.1]; 1033 women [82.2%] and 224 men [17.8%]; 249 Black or African American patients [19.8%], 990 White patients [78.8%], and 18 patients of other race or ethnicity [1.4%]) and 1809 patients treated with GLP-1RA (mean [SD] age, 45.4 [11.3] years; mean [SD] baseline BMI, 41.0 [7.9]; 1457 women [80.5%] and 352 men [19.5%]; 462 Black or African American patients [25.5%], 1231 White patients [68.0%], and 116 patients of other race or ethnicity [6.4%]). Diabetes was more common in the surgery group than in the GLP-1RA group (435 [34.6%] vs 531 [29.4%]), whereas hypertension and dyslipidemia were more common in the GLP-1RA group than in the surgery group (hypertension, 1342 [74.2%] vs 880 [70.0%]; dyslipidemia, 866 [47.9%] vs 531 [42.2%]). Within the surgery group, 734 (58.4%) underwent Roux-en-Y gastric bypass and 523 (41.6%) had sleeve gastrectomy. Among the patients in the GLP-1RA group, 1646 (91.0%) were treated with semaglutide and 163 (9.0%) with tirzepatide. At baseline, the GLP-1RA group had a higher mean (SD) FFM to FM ratio than the surgery group (1.2 [0.5] vs 1.0 [0.3]).

**Table 1.** Baseline Characteristics of Study Patients

Characteristic	No. (%)		*P* value
	Bariatric surgery (n = 1257)	GLP-1RAs (n = 1809)	
Age, mean (SD), y	43.4 (10.3)	45.4 (11.3)	<.001
Sex			
Female	1033 (82.2)	1457 (80.5)	.27
Male	224 (17.8)	352 (19.5)	
Race			
Black or African American	249 (19.8)	462 (25.5)	<.001
White	990 (78.8)	1231 (68.0)	
Other^a^	18 (1.4)	116 (6.4)	
Treatment type			
RYGB	734 (58.4)	NA	NA
SG	523 (41.6)	NA	
Semaglutide	NA	1646 (91.0)	
Tirzepatide	NA	163 (9.0)	
Diabetes	435 (34.6)	531 (29.4)	.002
Hypertension	880 (70.0)	1342 (74.2)	.01
Dyslipidemia	531 (42.2)	866 (47.9)	.002
Baseline BMI, mean (SD)	46.8 (7.1)	41.0 (7.9)	<.001
Fat mass, mean (SD), kg	68.3 (17.2)	56.1 (22.0)	<.001
Fat-free mass, mean (SD), kg	63.3 (12.2)	60.6 (26.9)	.001
FFM to FM ratio, mean (SD)	1.0 (0.3)	1.2 (0.5)	<.001

Abbreviations: BMI, body mass index (calculated as weight in kilograms divided by height in meters squared); FFM, fat-free mass; FM, fat mass; GLP-1RA, glucagon-like peptide-1 receptor agonist; NA, not applicable; RYGB, Roux-en-Y gastric bypass; SG, sleeve gastrectomy.

^a^ Included American Indian or Alaska Native, Asian, Middle Eastern or North African, Native Hawaiian or Other Pacific Islander, multiracial, and unknown.

After both treatments, FM decreased significantly over time, with the patients in the surgery group showing a more marked FM reduction than the patients in the GLP-1RA group (Figure). The covariate-adjusted mean relative reductions in FM for the surgery group were 42.4% (95% CI, 41.5%-43.2%) at 6 months, 49.7% (95% CI, 48.8%-50.6%) at 12 months, and 49.7% (95% CI, 47.8%-51.5%) at 24 months; the covariate-adjusted mean relative reductions in FM for the GLP-1RA group were 10.3% (95% CI, 9.5%-11.0%) at 6 months, 17.3% (95% CI, 16.5%-18.1%) at 12 months, and 18.0% (95% CI, 16.4%-19.7%) at 24 months (Table 2). FFM was also reduced over time in both groups, with greater reductions observed in the surgery group than in the GLP-1RA group (Figure). The adjusted mean relative reductions in FFM for the surgery group were 7.8% (95% CI, 7.2%-8.4%) at 6 months, 10.6% (95% CI, 10.0%-11.2%) at 12 months, and 11.7% (95% CI, 10.4%-12.9%) at 24 months; the adjusted mean relative reductions in FFM for the GLP-1RA group were 1.8% (95% CI, 1.3%-2.4%) at 6 months, 3.0% (95% CI, 2.4%-3.5%) at 12 months, and 3.3% (95% CI, 2.1%-4.4%) at 24 months (Table 2). The mean FFML%TWL in the surgery group was 17.6% (95% CI, 16.5%-18.7%) at 6 months and 18.6% (95% CI, 17.7%-19.6%) at 12 months, and the mean FFML%TWL in the GLP-1RA group was 29.8% (95% CI, 26.3%-33.3%) at 6 months and 24.8% (95% CI, 21.3%-28.3%) at 12 months. The FFM to FM ratio increased significantly in both groups (Figure), with the surgery group maintaining a higher ratio than the GLP-1RA group throughout 24 months after treatment. The FFM to FM ratios in the surgery group were 1.8 (95% CI, 1.8-1.8) at 6 months, 2.1 (95% CI, 2.1-2.1) at 12 months, and 2.0 (95% CI, 2.0-2.1) at 24 months, and the FFM to FM ratios in the GLP-1RA group were 1.4 (95% CI, 1.4-1.4) at 6 months, 1.5 (95% CI, 1.4-1.5) at 12 months, and 1.5 (95% CI, 1.5-1.6) at 24 months (Table 2).

**Figure.**
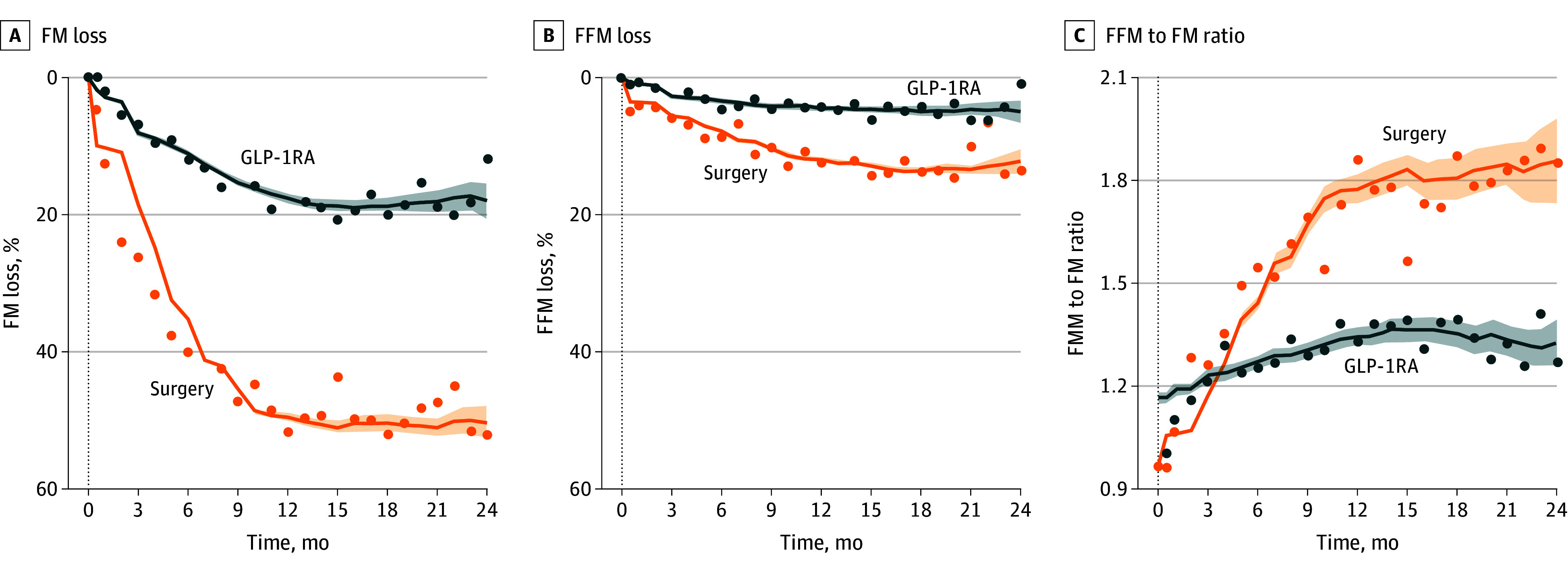
Longitudinal Changes in Body Composition After Bariatric Surgery or Glucagon-Like Peptide-1 Receptor Agonist (GLP-1RA) Treatment For months with numbers of observations greater than 10, mean values at each month are shown as points. Smoothed trajectories used a centered rolling window (width = 6 months), and 95% CIs (shaded areas) were calculated using pooled-variance standard errors across the window. FFM indicates fat-free mass; FM, fat mass.

**Table 2.** Changes in Body Composition After Bariatric Surgery or GLP-1RA Treatment

Body composition measurement	Baseline, mean	Least-squares mean value (95% CI)^a^		
		At 6 mo	At 12 mo	At 24 mo
FM relative reduction, %				
Bariatric surgery	0.0	42.4 (41.5-43.2)	49.7 (48.8-50.6)	49.7 (47.8-51.5)
GLP1-RA	0.0	10.3 (9.5-11.0)	17.3 (16.5-18.1)	18.0 (16.4-19.7)
FFM relative reduction, %				
Bariatric surgery	0.0	7.8 (7.2-8.4)	10.6 (10.0-11.2)	11.7 (10.4-12.9)
GLP1-RA	0.0	1.8 (1.3-2.4)	3.0 (2.4-3.5)	3.3 (2.1-4.4)
FFM to FM ratio				
Bariatric surgery	1.0	1.8 (1.8-1.8)	2.1 (2.1-2.1)	2.0 (2.0-2.1)
GLP1-RA	1.2	1.4 (1.4-1.4)	1.5 (1.4-1.5)	1.5 (1.5-1.6)

Abbreviations: FFM, fat-free mass; FM, fat mass; GLP-1RA, glucagon-like peptide-1 receptor agonist.

^a^ Marginal and conditional *R*^2^ values were 0.679 and 0.789 for FM, 0.214 and 0.502 for FFM, and 0.462 and 0.638 for the FFM to FM ratio, respectively. *P* < .001 for the differences between the surgery and GLP1-RA groups at 6, 12, and 24 months.

Similar trends of significant reductions in FM and increases in FFM to FM ratio were observed in patient subgroups defined by sex, race, baseline BMI, history of diabetes, and GLP-1RA treatment duration (eFigures 2-6 in Supplement 1 show observed body composition changes over time in each subgroup; eTable 1 in Supplement 2 shows the number of patients contributing to each time bin by treatment and subgroup; and eTables 2-6 in Supplement 2 show adjusted mean results at 6, 12, and 24 months in each patient subgroup with ≥10 patients). However, FFM changes seemed to vary by sex. Male patients had no significant FFM reductions after GLP-1RA treatment or bariatric surgery at 6 months and maintained this FFM preservation through 12 to less than 24 months after GLP-1RA treatment (eFigure 2 in Supplement 1 and eTable 2 in Supplement 2). In contrast, female patients experienced significant reductions in FFM after both GLP-RA treatment and bariatric surgery. The mean FFML%TWL was also higher among female than male patients, especially after surgery (19.6% [95% CI, 18.8%-20.5%] vs 7.4% [95% CI, 3.0%-11.8%] at 6 months after surgery, and 19.9% [95% CI, 19.2%-20.7%] vs 10.6% [95% CI, 5.6%-15.6%] at 12 months after surgery; 29.9% [95% CI, 26.5%-33.3%] vs 29.4% [95% CI, 16.5%-42.4%] at 6 months after GLP-1RA treatment and 25.3% [95% CI, 21.9%-28.7%] vs 22.0% [95% CI, 7.83%-36.3%] at 12 months after GLP-1RA treatment). Nevertheless, increases in the FFM to FM ratio were significant in both male and female patients, particularly after bariatric surgery.

## Discussion

In this single-center, retrospective cohort study of patients treated with current GLP-1RAs (semaglutide or tirzepatide) or bariatric surgery, we found that both treatments were associated with significant reductions in FM and FFM. Bariatric surgery was associated with greater relative reductions in both FFM and FM, as well as a significantly increased FFM to FM ratio. Male patients showed better preservation of FFM than female patients, especially after GLP-1RA treatment.

Bariatric surgery and GLP-1RA medications are currently the most effective weight-loss interventions for people with obesity, especially for those with severe obesity and comorbidities such as type 2 diabetes, sleep apnea, dyslipidemia, and CVD.^31,32,33,34^ Several studies have compared body weight and BMI after bariatric surgery or GLP-1RA treatment.^21,22,23,24^ A 2022 meta-analysis of 2 randomized clinical trials (RCTs) and 3 observational studies (total n = 332) estimated a 22.7- to 25.1-kg greater weight loss with surgery than with GLP-1RAs.^21^ A recent analysis found consistently greater TWL over 2 years after bariatric surgery (28.3% [n = 1291]) than after GLP-1RA treatment (10.3% [n = 257]).^22^ Studies with longer follow-up periods reported similar results. A 6.8-year study with more than 3000 matched pairs of patients with type 2 diabetes and obesity reported that surgery was associated with a 31.4% maximal BMI reduction and a 24.2% long-term BMI reduction compared with a 12.8% maximal BMI reduction and a 7.5% long-term BMI reduction for first-generation GLP-1RAs.^23^ Similarly, a 7.5-year study found mean maximal BMI reductions of 31.1% with surgery (n = 3178) vs 12.9% with GLP-1RAs (n = 3178).^24^

However, evaluating the effects of weight loss treatments solely by BMI would overlook the importance of body composition. Growing evidence suggests that FM and FFM may have distinct and often opposing effects on mortality.^35^ Among 1951 healthy Danish adults followed up over 18 years, greater FM and lower FFM were each independently associated with increased early mortality.^36^ Similarly, a 22-year prospective study of 787 older Swedish men found that FM percentage was positively associated with all-cause mortality, while FFM percentage was inversely associated with all-cause mortality.^37^ The Health Professionals Follow-up Study further supported these findings, reporting positive associations between predicted FM and mortality from all causes, CVD, and cancer, as well as a U-shaped association between predicted lean body mass (LBM) and mortality.^38^ The higher mortality observed at both low and high levels of LBM may be due to underlying conditions of underweight and overweight, as these measures were based on absolute mass rather than proportional composition. In addition, sarcopenic obesity, a condition characterized by a decrease in muscle mass and function and an increase in adipose tissue,^39^ is a growing concern, particularly among older adults. Sarcopenic obesity has been associated with elevated all-cause mortality, CVD incidence and mortality, and risks of metabolic syndrome, type 2 diabetes, dyslipidemia, and depressive symptoms.^40,41,42^ Collectively, these findings underscore the importance of obesity treatments that maximize FM reduction while maintaining and ideally enhancing the FFM to FM ratio.^43^

Our study supports that both bariatric surgery and GLP-1RAs may result in significant reductions in FM and FFM but are nonetheless beneficial due to the proportionally greater loss in FM.^39^ Previous meta-analyses have reported approximately 30-kg reductions in FM and approximately 13.5% decreases in body fat 12 months after bariatric surgery.^44,45^ Meanwhile, reductions in FFM were estimated at approximately 8.2 kg (95% CI, −10.7 to −5.7 kg) at 12 months, most of which occurred within the first 3 months after surgery.^43^ For GLP-1RAs, existing evidence also consistently shows greater losses in FM than LBM.^46,47,48^ A meta-analysis of 18 RCTs (n = 1363) found that semaglutide was significantly associated with reduced FFM by 1.7 kg (95% CI, −2.8 to −0.5 kg) compared with placebo.^49^ Another network meta-analysis comprising 22 RCTs (n = 2258) reported that GLP-1RAs were associated with a mean FM loss of 3.0 kg and LBM loss of 0.9 kg over a median follow-up of 24 weeks, with LBM accounting for approximately 25% of the total weight loss,^25^ consistent with our estimates that mean FFML%TWL was approximately 25% at 12 months after GLP-1RA treatment. This network meta-analysis also reported that the relative LBM, defined as percentage change from baseline, remained unaffected.^25^ In a retrospective cohort of 94 individuals, the mean loss was 2.7 kg in FM, 1.4 kg in LBM, and 0.9 kg in skeletal muscle mass.^50^ These findings suggest that both interventions are associated with improved body composition by favoring fat loss over FFM loss, which confirms our findings that GLP-1RAs were associated with an increased FFM to FM ratio over time. To our knowledge, our study is the first to estimate longitudinal changes in body composition among patients using the current generation of GLP-1RAs outside of an RCT setting on a large scale.^51^ Our study also provided long-term estimates of relative reductions in FFM and FM, as well as their ratio, throughout the entire follow-up period, rather than only the maximal values, as in previous longitudinal observational studies on this topic.

In stratified analyses, we observed a potential sex difference in body composition changes, which echoes the established physiological differences—men generally have greater muscle mass, lower FM%, and denser bones than women.^52,53,54^ Estrogen contributes to fat distribution and energy regulation through its interaction with leptin, the melanocortin system, and inflammatory pathways.^55,56,57^ Moreover, women are more prone to losing LBM during caloric restriction, possibly due to lower anabolic hormone levels and reduced muscle protein synthesis in response to exercise or protein intake.^58,59^ Emerging evidence supports sex-specific adaptations to dietary and exercise interventions, such as women deriving less muscle-preserving benefit from standard protein recommendations during weight loss.^60^ These findings and ours underscore the importance of considering sex in study design, analysis, and reporting of research findings.^61^ Future interventions should consider tailored strategies for preserving FFM in women, such as increased protein intake, resistance training, or combined modalities, especially when paired with pharmacologic or surgical treatments for weight loss.^62^

### Strengths and Limitations

Our study adds a strong piece of clinical evidence to the literature on weight loss treatment and body composition changes, particularly on longitudinal changes after using the new generation of GLP-1RAs. The main strengths of our study include its large sample size (n = 3066) and a relatively long follow-up period. Future research directions could leverage large prospective cohort designs with extended follow-up to investigate whether combined strategies, such as pairing GLP-1RAs with bariatric surgery, increased protein intake, and resistance training, can enhance weight loss, preserve FFM, and improve health outcomes.^63^

This study also has several limitations. First, body composition was assessed using BIA, which, while practical and noninvasive, is less precise than DXA, computed tomography, or magnetic resonance imaging.^64^ BIA’s accuracy can be influenced by hydration status, recent food or water intake, physical activity, and ambient temperature, potentially leading to measurement variability.^29^ In addition, BIA estimates FFM without distinguishing between its components, such as skeletal muscle, bone, organ tissue, and total body water, limiting the specificity of our findings.^65^ Nonetheless, evidence showed that BIA measurements had strong correlations with those acquired from DXA or magnetic resonance imaging.^30,66^ Second, our analysis of GLP-1RAs did not account for variations in dosage or patient adherence. Clinical data indicate that adherence to GLP-1RA treatment is suboptimal, with discontinuation rates reaching up to 70% within 2 years, often due to adverse effects, cost, or patient preferences.^67,68^ To address this limitation, we included only patients who had at least 2 GLP-1RA prescriptions or had 5% or more weight loss, to identify those with likely continued medication use and good adherence. We also conducted a stratified analysis by GLP-1RA treatment duration and found similar trends in body composition changes over 12 to 24 months. Third, the retrospective design inherently carries potential selection bias, heterogeneity between the bariatric surgery and GLP-1RA patient groups, and unmeasured confounding.^69^ Although we controlled for major covariates (eg, age, sex, race, baseline BMI, diabetes status, and treatment year in our generalized linear mixed models) and conducted stratified analyses by these factors, heterogeneity and residual confounding could not be ruled out, making the 2 treatment groups not directly comparable. Nevertheless, our study was not designed for direct comparisons between the 2 groups. Fourth, given the relatively small sample size in certain subgroups (eg, men or Black patients), model fit and generalizability may be limited, and the findings should be interpreted with caution. Fifth, we did not account for lifestyle factors, such as diet and physical activity, or other potential weight management programs, due to the lack of data in the EHR. We also did not evaluate the associations between changes in body composition and clinical health outcomes, such as metabolic improvements or quality of life measures. Understanding these associations is crucial for assessing the clinical significance of body composition changes.^70^

## Conclusions

In this single-center cohort study of 3066 patients, both bariatric surgery and current GLP1-RAs (semaglutide and tirzepatide) were associated with significant reductions in FM, modest reductions in FFM, and increases in the FFM to FM ratio over 24 months. These trends were generally consistent across key subgroups defined by sex, race, baseline BMI, diabetes history, and treatment duration. Overall, our findings indicate a favorable shift in body composition after surgical or medical weight loss, providing evidence to inform clinical obesity care and interventions aimed at preserving FFM while promoting fat loss during obesity treatment.

## Notes

Supplement 1. **eFigure 1.** Study Flowchart **eFigure 2.** Body Composition Changes After Bariatric Surgery or GLP1-RA Treatment by Sex **eFigure 3.** Body Composition Changes After Bariatric Surgery or GLP1-RA Treatment by Race **eFigure 4.** Body Composition Changes After Bariatric Surgery or GLP1-RA Treatment by Baseline BMI **eFigure 5.** Body Composition Changes After Bariatric Surgery or GLP1-RA Treatment by Diabetes History **eFigure 6.** Body Composition Changes After Bariatric Surgery or GLP1-RA Treatment by GLP-1RA Duration

Supplement 2. **eTable 1.** Number of Participants Contributing to Each Time Bin by Treatment and Stratification **eTable 2.** Changes in Body Composition Following Bariatric Surgery or GLP-1RA Treatment by Sex **eTable 3.** Changes in Body Composition Following Bariatric Surgery or GLP-1RA Treatment by Race **eTable 4.** Changes in Body Composition Following Bariatric Surgery or GLP-1RA Treatment by BMI ≤40 or >40 kg/m^2^ **eTable 5.** Changes in Body Composition Following Bariatric Surgery or GLP-1RA Treatment by Diabetes History **eTable 6.** Changes in Body Composition Following Bariatric Surgery or GLP-1RA Treatment for <12 or ≥12 Months

Supplement 3.
Data Sharing Statement
