# Supplementary material for: Body Composition Changes After Bariatric Surgery or Treatment With GLP-1 Receptor Agonists
Source: JAMA Netw Open. 2026 Jan 9;9(1):e2553323. doi: 10.1001/jamanetworkopen.2025.53323 (PMC12789952; doi:10.1001/jamanetworkopen.2025.53323)
Supplement: Supplement 1. — eFigure 1. Study Flowchart eFigure 2. Body Composition Changes After Bariatric Surgery or GLP1-RA Treatment by Sex eFigure 3. Body Composition Changes After Bariatric Surgery or GLP1-RA Treatment by Race eFigure 4. Body Composition Changes After Bariatric Surgery or GLP1-RA Treatment by Baseline BMI eFigure 5. Body Composition Changes After Bariatric Surgery or GLP1-RA Treatment by Diabetes History eFigure 6. Body Composition Changes After Bariatric Surgery or GLP1-RA Treatment by GLP-1RA Duration [file jamanetwopen-e2553323-s001.pdf]

# Supplementary Online Content

Wang Z, Wang L, Zhang X, et al. Body composition changes after bariatric surgery or treatment with GLP-1 receptor agonists. *JAMA Netw Open.* 2026;9(1):e2553323. doi:10.1001/jamanetworkopen.2025.53323

**eFigure 1.** Study Flow Chart

**eFigure 2.** Body Composition Changes After Bariatric Surgery or GLP1-RA Treatment by Sex

**eFigure 3.** Body Composition Changes After Bariatric Surgery or GLP1-RA Treatment by Race

**eFigure 4.** Body Composition Changes After Bariatric Surgery or GLP1-RA Treatment by Baseline BMI

**eFigure 5.** Body Composition Changes After Bariatric Surgery or GLP1-RA Treatment by Diabetes History

**eFigure 6.** Body Composition Changes After Bariatric Surgery or GLP1-RA Treatment by GLP-1RA Duration

This supplementary material has been provided by the authors to give readers additional information about their work.

**eFigure 1.** *Study flow chart*

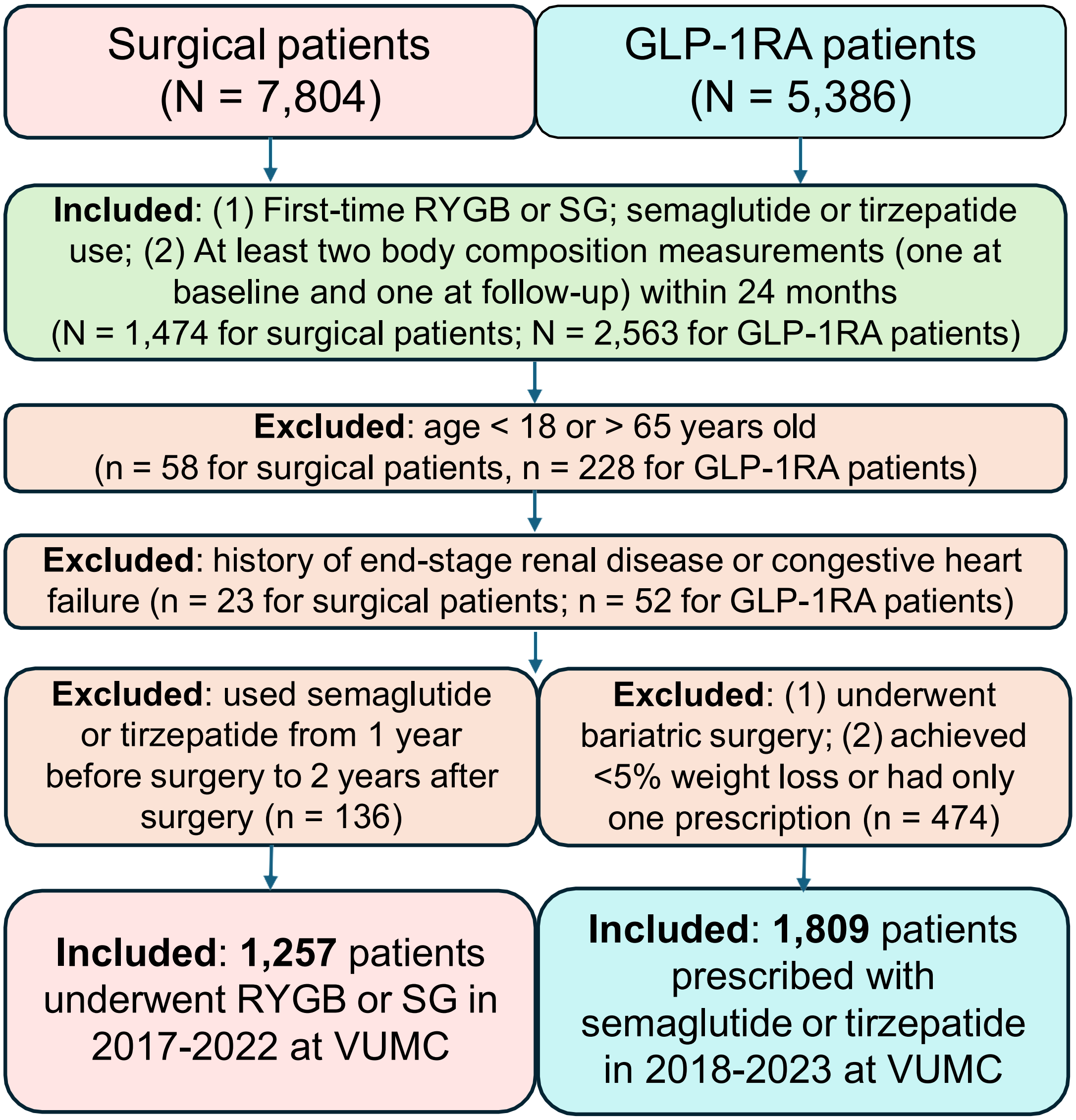


Abbreviations: GLP-1RAs, GLP-1 receptor agonists; RYGB, Roux-en-Y gastric bypass; SG, sleeve gastrectomy; VUMC, Vanderbilt University Medical Center.

© 2026 Wang Z et al. *JAMA Network Open.*

**eFigure 2.** *Body composition changes after bariatric surgery or GLP1-RA treatment by sex*[a]

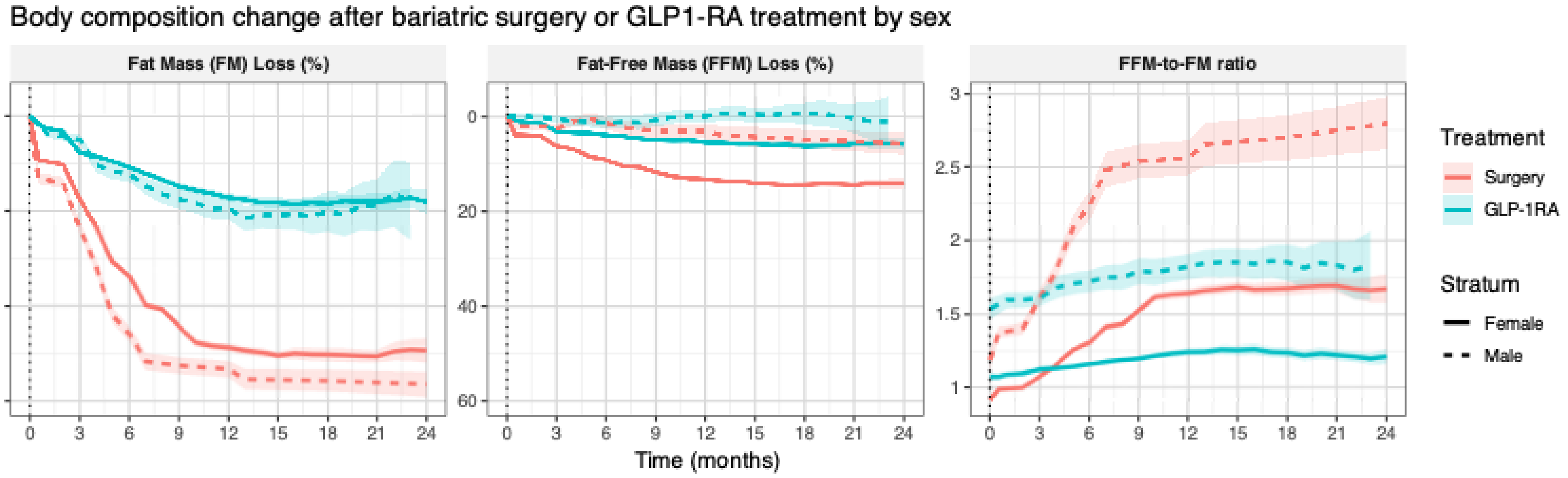


[a]Smoothed trajectories used a centered rolling window (width = 6 months) of mean values, and 95% CIs were calculated using pooled-variance standard errors across the window.

Abbreviations: FFM, fat-free mass; FM, fat mass; GLP-1RAs, GLP-1 receptor agonists; 95% CI, 95% confidence interval.

© 2026 Wang Z et al. *JAMA Network Open.*

**eFigure 3.** *Body composition changes after bariatric surgery or GLP1-RA treatment by race*[a]

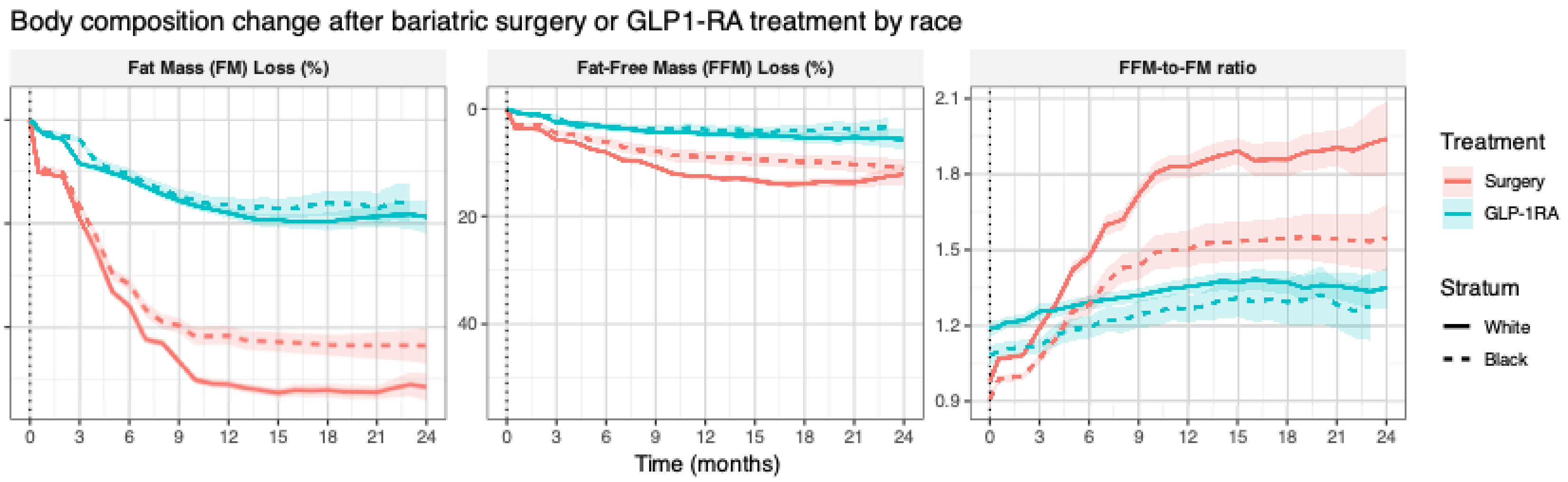


[a]Smoothed trajectories used a centered rolling window (width = 6 months) of mean values, and 95% CIs were calculated using pooled-variance standard errors across the window.

Abbreviations: FFM, fat-free mass; FM, fat mass; GLP-1RAs, GLP-1 receptor agonists; 95% CI, 95% confidence interval.

© 2026 Wang Z et al. *JAMA Network Open.*

**eFigure 4.** *Body composition changes after bariatric surgery or GLP1-RA treatment by baseline BMI*[a]

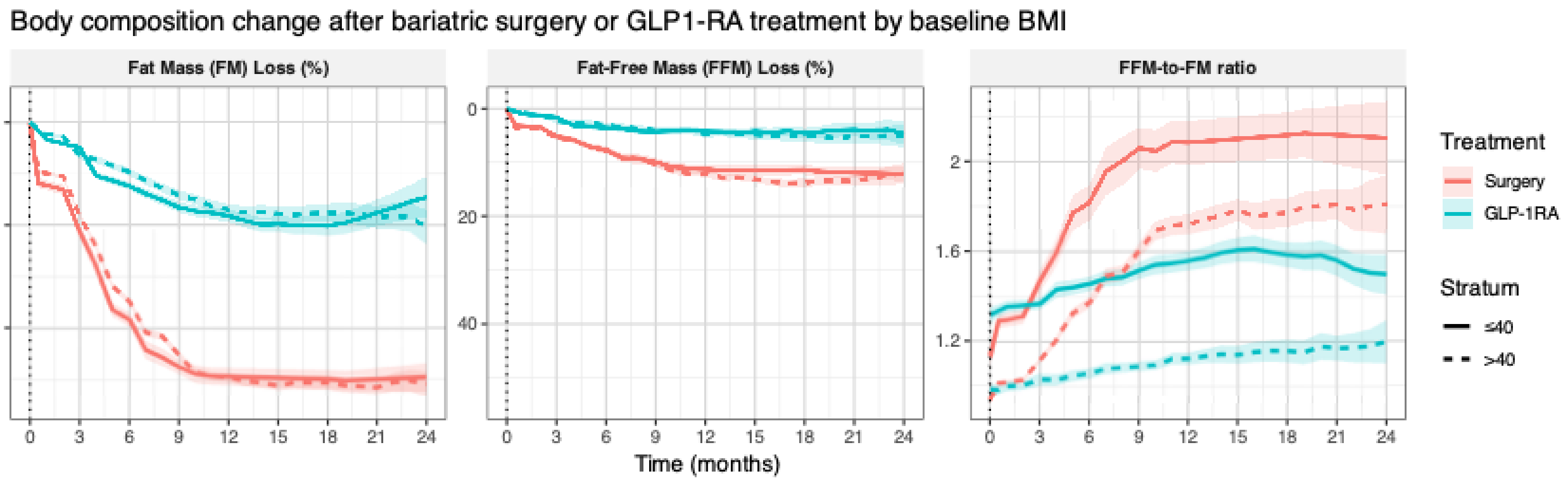


[a]Smoothed trajectories used a centered rolling window (width = 6 months) of mean values, and 95% CIs were calculated using pooled-variance standard errors across the window.

Abbreviations: FFM, fat-free mass; FM, fat mass; GLP-1RAs, GLP-1 receptor agonists; 95% CI, 95% confidence interval.

© 2026 Wang Z et al. *JAMA Network Open.*

**eFigure 5.** *Body composition changes after bariatric surgery or GLP1-RA treatment by diabetes history*[a]

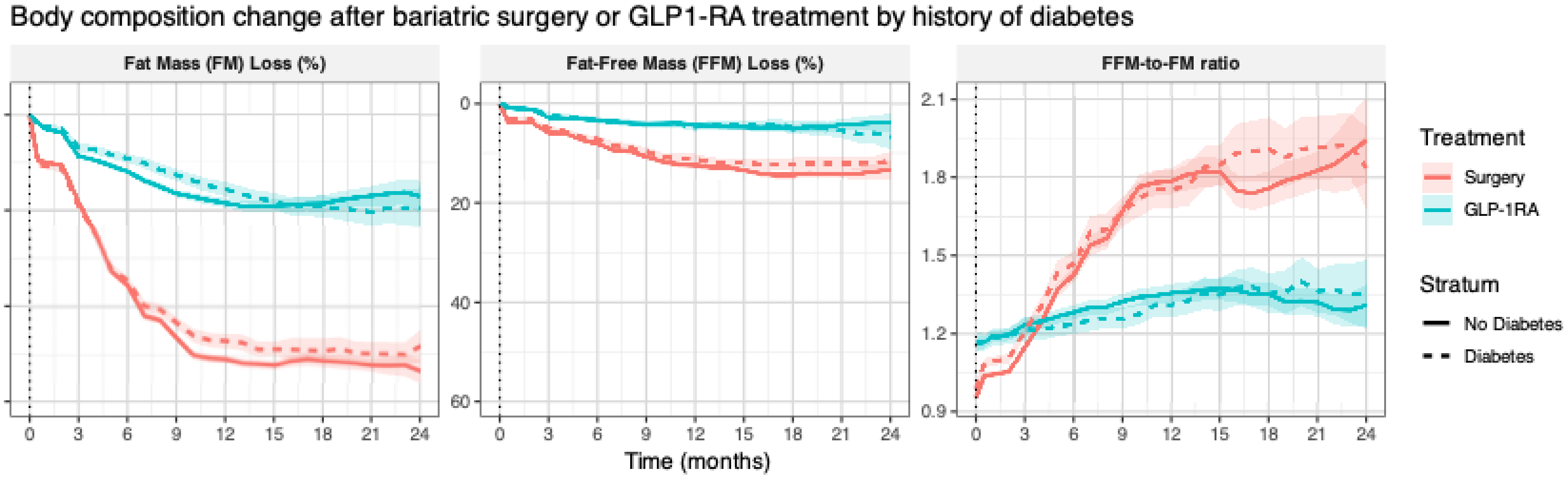


[a]Smoothed trajectories used a centered rolling window (width = 6 months) of mean values, and 95% CIs were calculated using pooled-variance standard errors across the window.

Abbreviations: FFM, fat-free mass; FM, fat mass; GLP-1RAs, GLP-1 receptor agonists; 95% CI, 95% confidence interval.

© 2026 Wang Z et al. *JAMA Network Open.*

**eFigure 6.** *Body composition changes after bariatric surgery or GLP1-RA treatment by GLP-1RA duration*[a]

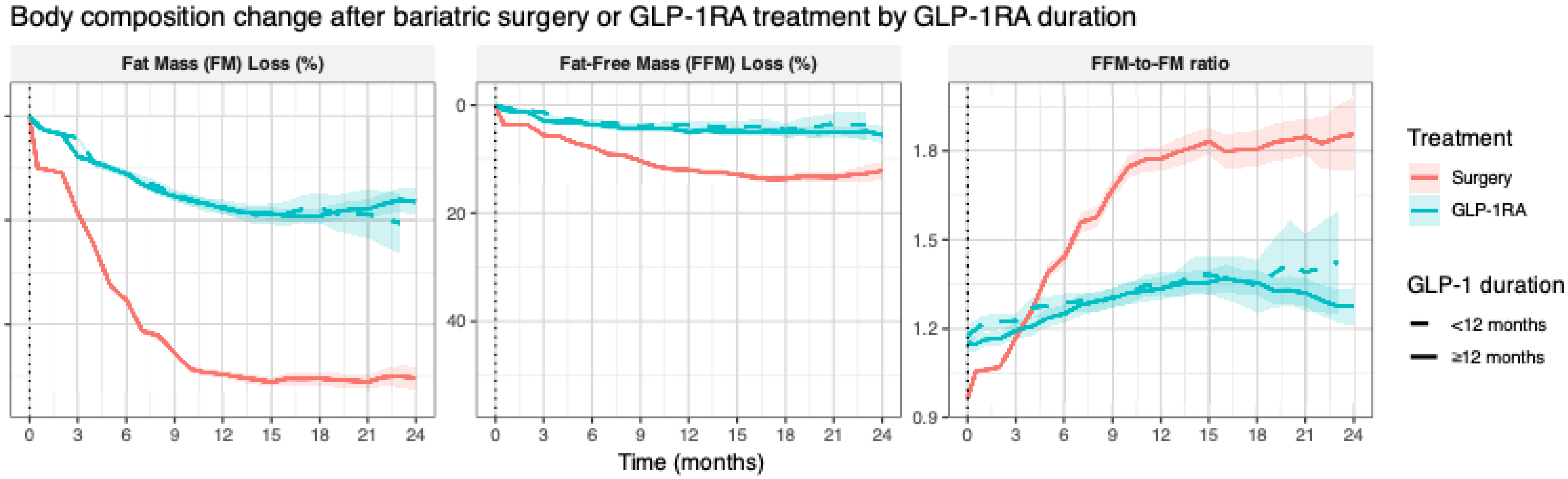


[a]Smoothed trajectories used a centered rolling window (width = 6 months) of mean values, and 95% CIs were calculated using pooled-variance standard errors across the window.

Abbreviations: FFM, fat-free mass; FM, fat mass; GLP-1RAs, GLP-1 receptor agonists; 95% CI, 95% confidence interval.

© 2026 Wang Z et al. *JAMA Network Open.*
