# Supplementary material for: Body Composition Changes After Bariatric Surgery or Treatment With GLP-1 Receptor Agonists
Source: JAMA Netw Open. 2026 Jan 9;9(1):e2553323. doi: 10.1001/jamanetworkopen.2025.53323 (PMC12789952; doi:10.1001/jamanetworkopen.2025.53323)
Supplement: Supplement 3. — Data Sharing Statement [file jamanetwopen-e2553323-s003.pdf]

Data Sharing Statement

Wang. Body Composition Changes After Bariatric Surgery or Treatment With GLP-1 Receptor Agonists. *JAMA Netw Open*. Published January 09, 2026.
doi:10.1001/jamanetworkopen.2025.53323

Data
Data available: No

Additional Information
Explanation for why data not available: The datasets generated during and/or analyzed during the current study are not publicly available to protect patient confidentiality.
